# A novel prognostic marker and immunogenic membrane antigen: prohibitin (PHB) in pancreatic cancer

**DOI:** 10.1038/s41424-018-0044-1

**Published:** 2018-09-06

**Authors:** Weibin Wang, Lai Xu, Yu Yang, LiangBo Dong, BangBo Zhao, Jun Lu, Taiping Zhang, Yupei Zhao

**Affiliations:** 1 Department of General Surgery, Peking Union Medical College Hospital, Chinese Academy of Medical Science & Peking Union Medical College, 1 Shuai Fu Yuan Hu Tong, Beijing, 100730 China; 2 Department of General Surgery, Beijing Tsinghua Chang Gung Hospital, Tsinghua University, 168 soup road, Changping District, Beijing, 102218 China

## Abstract

**Background:**

Previous study, using immunoblotting with IgG and membrane proteins, identified prohibitin (PHB) as a potential immunogenic membrane antigen. Now, investigate PHB expression and biological functions in pancreatic cancer.

**Methods:**

PHB expression was analysed in PDAC cell lines, normal pancreas tissues, cancer tissues, PDAC patient sera and healthy volunteer sera using QRT-PCR, Western blotting, IHC, and ELISA, and a survival analysis and a COX regression analysis were performed. Low and high PHB expression levels were accomplished using RNA interference technology and gene transfer techniques. Cell proliferation, migration, and invasion, apoptosis-related proteins were assessed 48 h after transfection.

**Results:**

PHB was generally expressed in the 8 tested PDAC cell lines. PHB was significantly increased in PDAC tissues and negatively correlated with overall survival (*p* < 0.01). PHB was an independent prognostic factor in PDAC (*p* < 0.01). PHB was increased in PDAC patient sera (*p* < 0.01). siRNA-PHB decreased cell growth, migration and invasion. However, PHB overexpression resulted in the opposite effects. Western blotting and Flow cytometric analysis revealed apoptosis inhibition in siRNA-PHB PDAC cells.

**Conclusions:**

PHB plays a key role in modulating the malignant phenotype and apoptosis induction, which may be a novel prognostic predictor and a candidate for targeted therapy against PDAC.

## Introduction

Pancreatic cancer is one of the most aggressive clinical malignancies, and it remains the fourth leading cause of cancer-related deaths. In contrast to the steady increase in survival for most cancers, advances have been slow for pancreatic cancers, and the 5-year relative survival is currently 8%. These low survival rates partially occur because more than one-half of cases are diagnosed at a distant stage^1^. Chemotherapy, such as Gemcitabine, also does not contribute a significant survival benefit^2–4^. Therefore, there is strong demand for novel specific markers in pancreatic cancer patients for early diagnosis, targeted therapy and prognosis.

Autoimmunity was demonstrated against several proteins, including MUC1, p53, and Rad51, in pancreatic cancer. MUC1 autoantibodies were observed in sera from patients with a variety of different tumours and have been associated with a favourable prognosis in pancreatic cancer^5^. The presence of p53 autoantibodies has been observed in 18.2% of patients with pancreatic cancer but was not specific to malignancy^6,7^. The recombination factor Rad51 is highly expressed in pancreatic adenocarcinoma, and Rad51 autoantibodies have been observed in 7% of patients with pancreatic cancer^8^. Membrane proteins associated with pancreatic cancer perform many essential cellular functions, and hopefully these proteins become more valuable molecular markers. A previous study identified autoantibodies of membrane proteins in pancreatic cancer using membrane biology, cellular component proteomic, immuno-proteomic, and membrane proteomic approaches, and prohibitin was noted as a potential immunogenic membrane antigen^9^.

Prohibitin (PHB), including PHB1 (BAP32), PHB2 (BAP37, REA), PHB3 and PHB4, is ubiquitously expressed in multiple cellular compartments, including the mitochondria, the nucleus and the plasma membrane, and PHB is shuttled between the mitochondria, the cytosol and the nucleus^10–12^. PHB contains an N-terminal transmembrane domain that is an evolutionarily conserved PHB domain similar to that in lipid raft-associated proteins and a C-terminal coiled-coil domain that is involved in the regulation of protein-protein interactions^13–15^. However, the role of PHB in different cancers is controversial, and the biological functions of PHB in pancreatic cancer is not clear. The current study investigated the differential expression of PHB in normal and pancreatic cancer patient sera, tissues and cancer cells; examined the relationship between PHB expression and clinicopathological variables; and explored the biological functions of PHB in cancer cell proliferation, migration, invasion and apoptosis.

## Materials and methods

### Cell culture

Human pancreatic cell lines, including AsPC-1, Capan-1, T3M4, SW1990, SU86.86, Panc-1, Colo-357, BxPC-3, and MiaPaCa-2, were kindly donated by Professor Helmut Freiss from the Technical University Munich of Germany. The MiaPaCa-2 human pancreatic cancer cell line was maintained in Dulbecco’s modified Eagle’s medium (DMEM), and AsPC-1 was maintained in RPMI 1640 medium. Both media were supplemented with 10% foetal bovine serum (FBS), and the cells were maintained at 37 °C in a humidified atmosphere of 5% CO_2_.

### Specific siRNA and transfection

The targeting sequences of a 25-nucleotide siRNA were designed and chemically synthesised (Invitrogen American): 5′-AGCACACGCUCAUCAUAGUCCUCUC-3′ (sense), and 3′-GAGAGGACUAUGAUGAGCGUGUGCU-5′ (antisense). Negative control siRNA (Invitrogen American) was used as a scrambled siRNA control. A total of 5 × 10^5^ cells were plated in a 6-well plate for 48 h, and siRNA transfection (120 pmol) was performed using the Lipofectamine 2000 (6 μl) (Invitrogen) according to the manufacturer’s instructions.

### Plasmid construction

The pMD18-T-PHB plasmid was digested with BamHI and EcoRI and ligated into the pIRES2-EGFP vector (Novagen, Madison, US). *E*. *coli* DH5α cells were transformed using the ligation mixture, and colonies were obtained. Presence of the PHB gene was examined using PCR amplification, and the products were stored for subsequent analysis.

### Western blot analysis

Proteins were extracted from the cells and subjected to SDS-PAGE. The proteins were transferred to polyvinylidene difluoride (PVDF) membranes (Millipore, USA). A polyclone rabbit anti-prohibitin antibody was purchased from Abcam (ab70672). Polyclonal rabbit anti-human PARP antibody, polyclonal rabbit anti-human caspase-3 antibody, polyclonal rabbit anti-human cleaved caspase-3 antibody, polyclonal rabbit anti-human caspase-9 antibody, polyclonal rabbit anti-human Bcl-2 antibody and polyclone rabbit anti-β-actin antibody were obtained from Cell Signaling Technology. The membranes were probed with all primary antibodies at a dilution of 1:1000, and secondary peroxidase-conjugated antibodies were used at a dilution of 1:2000. Bands were imaged using a chemiluminescence method (Amersham Biosciences, Sweden). Anti-β-actin antibody was used as an internal control.

### RNA extraction and quantitative real-time PCR (q-PCR)

Total RNA was extracted from the cells and tissues using TRIzol reagent (Invitrogen, CA, USA) according to the manufacturer’s instructions. cDNA was synthesised using M-MLV reverse transcriptase (Invitrogen) from 5 μg of total RNA. Quantitative RT-PCR was performed using a Bio-Rad CFX96 real-time PCR system (Bio-Rad, Foster City, CA, USA), KAPA PROBE FAST qPCR kits (Kapa Biosystems, MA, USA) and TaqMan probes (Invitrogen) with the following cycling conditions: 95 °C for 10 min (initial denature); 40 cycles of 95 °C for 15 s; and 60 °C for 60 s. The following sequences were used as PHB primers: 5′-GGGCACAGAGCTGTCATCTT-3′ and 5′-TGACTGGCACATTACGTGT-3′.

### Cell proliferation assay

The impact of PHB silencing on the proliferation of pancreatic cancer cells was measured using a Cell Counting Kit-8 (CCK-8) (Dojindo, Japan), according to the manufacturer’s instruction. A total of 1.0 × 10^3^ pancreatic cancer cells were added to each well of 96-well culture plates 24 h after transfection. Cell proliferation was assessed 0, 24, 48 and 72 h. CCK-8 reagent (10 μl) was added to each well, and the absorbance was measured at 450 nm after a 1.5-h incubation at 37 °C. All experiments were performed in 5 wells per experiment and repeated at least three times.

### In vitro migration/invasion assays

The migratory abilities of cells were evaluated using Transwell assays. Cell culture inserts with 8-μm microporous filters without extracellular matrix coating (Becton Dickinson Labware, Bedford, MA) were loaded with 200 μl of serum-free DMEM/RPMI 1640 containing 4.0 × 10^5^ pancreatic cancer cells. The bottom chambers were loaded with 500 μl of DMEM/RPMI 1640 containing 10% FBS. The cells on the lower surface of the filter after a 24-h incubation were fixed and stained. Five random optical fields (×100 magnifications) from triplicate filters were selected for quantification of migrated cells. The invasive abilities of the cells were also evaluated using Transwell assays but with extracellular matrix coating (Sigma).

### Apoptosis assay using Annexin V-FITC and propidium iodide (PI) staining

The rate of cell apoptosis was quantified by annexin-V–FITC and propidium iodide double staining using an Annexin-V/FITC kit (Neobiscience, China). Cells were collected according to the manufacturer’s instructions 48 h after transfection, washed with cold PBS, and suspended in binding buffer. The cells were incubated for 10 min in the dark at room temperature with Annexin V-FITC and PI in phosphate buffer and analysed using a flow cytometer (FACS CantoII, BD Bioscience, USA) within 1 h of staining.

### Caspase-3 activity assay

The effect of PHB on caspase-3 activity in AsPC-1 and MiaPaCa-2 cells was determined using a commercially available caspase-3 (active) ELISA kit (Applygen Technologies, China). An ELISA for caspase-3 activity was performed according to manufacturer’s instructions.

### Enzyme-linked immunosorbent assay (ELISA)

Sera from 31 pancreatic cancer patients and 31 healthy volunteers were obtained with the consent of patients and donors after approval from the Institutional Human Ethical Committee of the Peking Union Medical College Hospital, China. The human prohibitin ELISA kit (Life Sciences Advanced Technologies, China) was used to determine prohibitin levels according to manufacturer^’^s instruction.

### Immunohistochemical analysis

Immunohistochemistry (IHC) was performed to localise PHB expression in 10 normal and 66 pancreatic ductal adenocarcinoma (PDAC) samples. The PHB antibody was diluted 1:3000. Two pathologists who specialised in pancreatic cancer independently rated the staining intensity and percentage of stained cells. Briefly, scores were used to rate the staining intensity of cancer cells (no staining: 0; weak: 1; moderate: 2; and strong: 3) and determine the percentage of stained cells (<5%: (0); 5–25%: (1); >25–50%: (2); >50–75%: (3); and >75%: (4)). The final intensity score was equal to the staining intensity score multiplied by the cell percentage score. Staining was stratified accordingly into low levels of expression (scores <4) or high levels of expression (score ≥4).

### Statistical analysis

Statistical analysis was performed using SPSS23 software (SPSS, Inc., Chicago, IL). Median values were taken as cutoff limits when two groups were compared. Survival analyses were performed using the Kaplan–Meier method log-rank test. Multivariable analysis was performed using a Cox proportional hazards model. The median survival and estimations of hazard ratios were reported with 95% confidence intervals. Comparisons of demographic and clinicopathological data between groups were performed using a *Χ*^2^ test. The measurement data are presented as means ± SD and compared using Student’s t test or the Mann–Whitney *U* test. Statistical significance was set at a *P* value less than 0.05. GraphPad Prism 5 (GraphPad, San Diego, CA) software was used to create graphs.

## Results

### Prohibitin was expressed in PDAC cell lines, and prohibitin silencing inhibited the proliferation, migration and invasion of AsPC-1 and MiaPaCa-2 cells

**Fig. 1:**
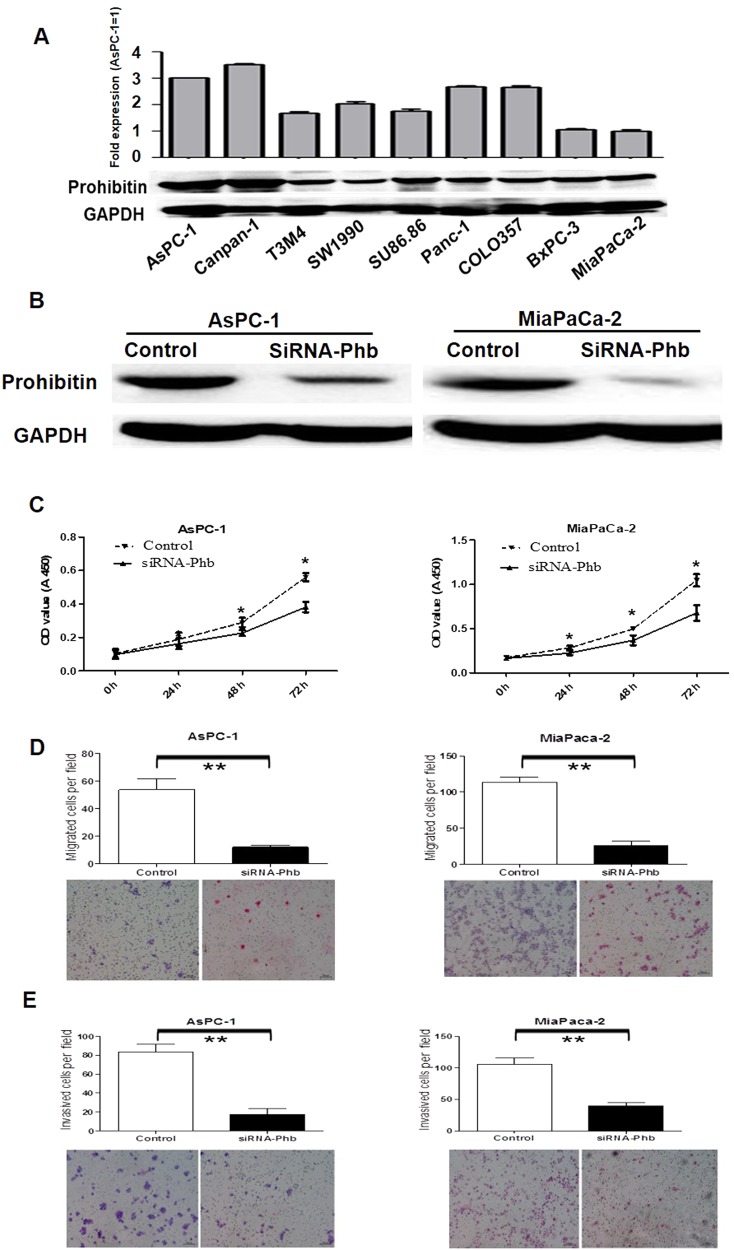
Prohibitin expression in pancreatic cancer cell lines, proliferative, migratory and invasive ability was significant reduced after siRNA-Phb transfection in AsPC-1 and MiaPaCa-2 cells. **a** Prohibitin mRNA and protein expression were generally expressed in 8 pancreatic cancer cell lines with Real-Time PCR and western blot. AsPC-1 and MiaPaCa-2 cells were selected for further functional experiments due to their various PHB expression. β-actin was used as housekeeping. GAPDH was used as a loading control. **b** AsPC-1 and MiaPaCa-2 cells were transfected with control siRNA or PHB siRNA for 48 h, total proteins were extracted and western blotting analysis was performed, 59.6% and 87.1% PHB proteins were reduced, respectively. **c** The effects of siRNA-PHB on pancreatic cancer cells AsPC-1 and MiaPaCa-2, the growth after 24 h, 48 h and 72 h were assessed using CCK-8 assay (mean ± SD; *p < 0.05). **d** The migration of AsPC-1 and MiaPaCa-2 cells were assessed after siRNA-PHB transfection and compared with negative control transfected cells (mean ± SD; **p < 0.01). **e** The invasiveness of AsPC-1 and MiaPaCa-2 cells were assessed after siRNA-PHB transfection and compared with negative control transfected cells (mean ± SD; **p < 0.01)

We investigated PHB expression in 8 pancreatic cancer cell lines, and PHB mRNA and protein were detectable in the cell lines. The AsPC-1 and MiaPaCa-2 cell lines were chosen for further functional experiments because of their high and low levels of PHB expression, respectively (Fig. 1a). We performed transient silencing of PHB in AsPC-1 and MiaPaCa-2 cells. Specific PHB RNA interference (siRNA-PHB) molecules resulted in 71.6 and 87.1% reductions of PHB expression in AsPC-1 and MiaPaCa-2 cells at 48 h, respectively (Fig. 1b). CCK-8 assays were performed to assess the effects of PHB silencing on the proliferation of AsPC-1 and MiaPaCa-2 cells. We observed a significant decrease in growth rate after effective reduction of endogenous PHB at 24, 48 and 72 h (*P* < 0.05) compared to the control cells (Fig. 1c). Transwell assays were performed to examine the potential role of PHB on cell migration and invasion. Knockdown of PHB expression significantly suppressed the migration of AsPC-1 (*P* < 0.01) and MiaPaCa-2 (*P* < 0.01) cells (Fig. 1d). The invasive abilities of siRNA-PHB AsPC-1 (*P* < 0.001) and MiaPaCa-2 (*P* < 0.001) cells were dramatically decreased compared to the control cells (Fig. 1e).

### Overexpression of the PHB gene in AsPC-1 and MiaPaCa-2 cells increased proliferation, migration and invasion

**Fig. 2:**
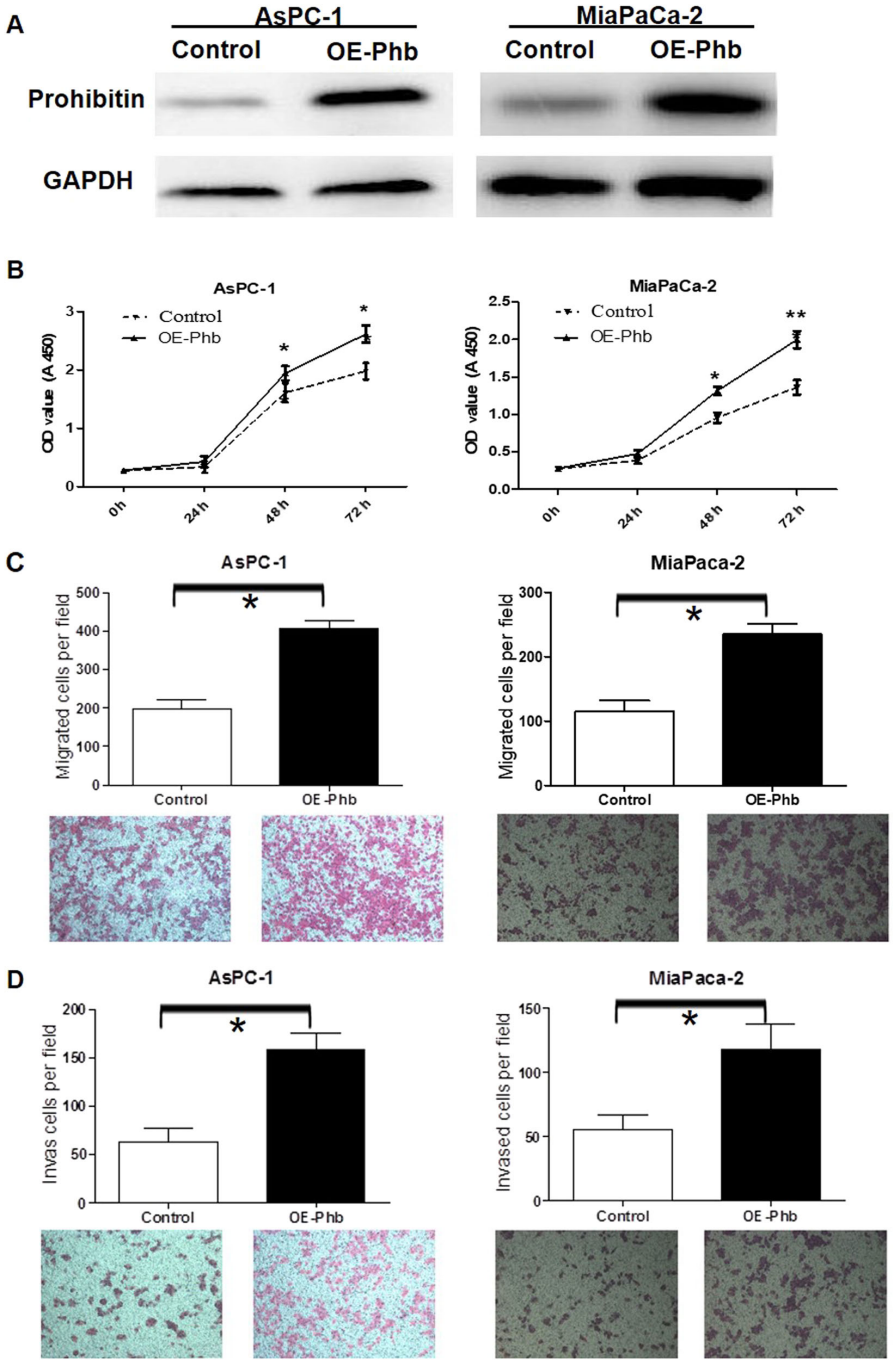
Effects of overexpressed PHB on pancreatic cancer cells AsPC-1 and MiaPaCa-2 proliferation, migration and invasion. **a** Overexpressed PHB plasmids were transfected into AsPC-1 and MiaPaCa-2 cells for 48 h, total proteins were extracted and Western Blotting analysis was performed, 81.3 and 90.6% PHB prtoteins were overexpressed, respectively. **b** The effects of PHB overexpressed on pancreatic cancer cells AsPC-1 and MiaPaCa-2, the growth after 24, 48 and 72 h were assessed using CCK-8 assay (mean ± SD; *p < 0.05, **p < 0.01). **c** The migration of AsPC-1 and MiaPaCa-2 cells were assessed after overexpressed PHB plasmid transfection and compared with negative control transfected cells (mean ± SD; *p < 0.05). **d** The invasiveness of AsPC-1 and MiaPaCa-2 cells were assessed after overexpressed PHB transfection and compared with negative control transfected cells (mean ± SD; *p < 0.05)

AsPC-1 and MiaPaCa-2 cells were transfected with PHB to confirm the effect of siRNA-PHB on cells proliferation, migration and invasion. Specific pIERS2-EGFP-PHB transfection resulted in 81.3% and 90.6% overexpression of PHB in AsPC-1 and MiaPaCa-2 cells at 48 h, respectively, according to Western blotting analysis (Fig. 2a). CCK-8 assays were performed to assess the effects of PHB overexpression on proliferation. An obvious increase in growth rate was observed after effective PHB transfection at 24, 48 and 72 h in AsPC-1 (*P* < 0.05) and MiaPaCa-2 (*P* < 0.05) cells compared to the control cells (Fig. 2b). Transwell assays were performed to examine the impact of PHB overexpression on cell migration and invasion abilities. PHB overexpression significantly increased the migration ability of AsPC-1 (*P* < 0.05) and MiaPaCa-2 (*P* < 0.05) cells (Fig. 2c). PHB overexpression distinctly increased the invasive abilities of AsPC-1 (*P* < 0.05) and MiaPaCa-2 (*P* < 0.05) cells compared to controls (Fig. 1d).

### Ablation of PHB expression reduced apoptosis in PDAC cells

**Fig. 3:**
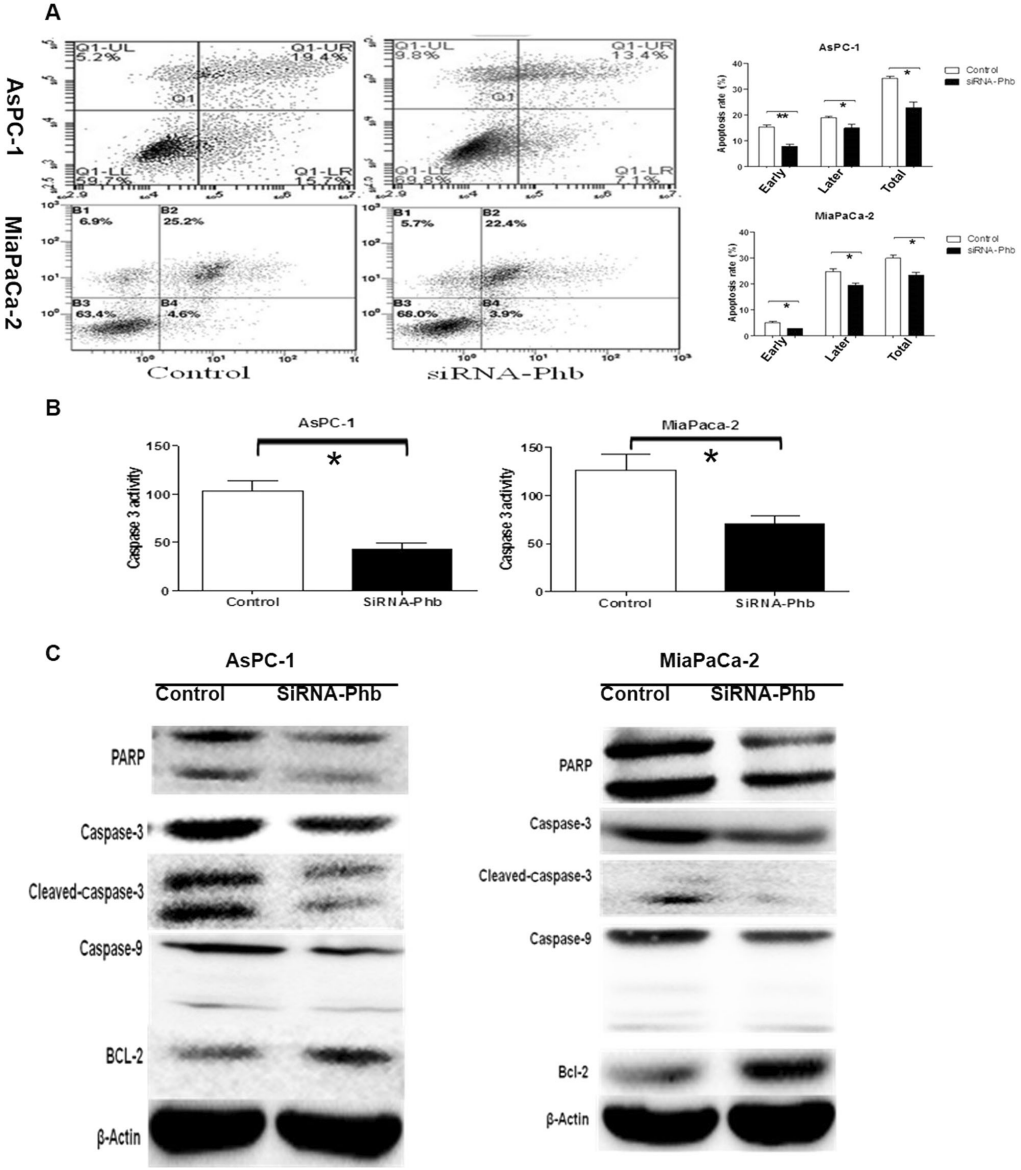
siRNA-PHB inhibited apoptosis in pancreatic cancer cells. **a** After siRNA-PHB transfection 48h, the cells were harvested and double stained with Annexin-V and PI. The early, later and total apoptosis rate of siRNA-PHB cells was obviously lower than the control cells in AsPC-1 and MiaPaCa-2. (*p < 0.05, **p < 0.01). **b** After siRNA-PHB transfection 48h, the protease activity of Caspase-3 notably decreased in AsPC-1 cells and in MiaPaCa-2 cells compared with the control, respectively. (*p < 0.05). **c** Knockdown PHB resulted in a statistically significant decrease in the expression of PARP, caspase-3, cleaved caspase-3 and caspase-9 protein in AsPC-1 (*p* < 0.05) and MiaPaCa-2 (*p* < 0.05) cells, compared to the control group, respectively. However, the expression of Bcl-2 was significantly increased in the AsPC-1 (*p* < 0.05) and MiaPaCa-2 (*p* < 0.01) cells after siRNA-PHB transfection 48 h

Annexin-V and PI staining assays were used with flow cytometry to quantify the effect of siRNA-PHB on apoptosis in human pancreatic cancer cells. Cells were stained with Annexin V-FITC/PI and gated into the lower right (LR) and upper right (UR) quadrants (Fig. 3). Cells in the LR and UR quadrants were considered early apoptotic (Annexin^+^/PI^−−^) and late apoptotic (Annexin^+^/PI^+^), respectively. Cells in the LL (lower left) and UL (upper left) quadrants were considered alive and necrotic, respectively. The extent of apoptosis is expressed as the sum of the percentages in the LR and UR quadrants. Cells treated with siRNA-PHB exhibited a decrease in apoptosis of 22.8% (siRNA-PHB) to 34.3% (negative control) in AsPC-1 cells (*p* < 0.01) and from 26.37% (siRNA-PHB) to 29.87% (negative control) in MiaPaCa-2 cells (*p* < 0.05) (Fig. 3a). The caspase cascade and several closely related mitochondrial apoptosis proteins were analysed in AsPC-1 and MiaPaCa-2 cells to elucidate the possible pathway of PHB-induced apoptosis. The protease activity of caspase-3 48 h after PHB RNA interference decreased notably in AsPC-1 cells (*p* < 0.01) and MiaPaCa-2 cells (*p* < 0.01) compared to the controls cells (Fig. 3b). PHB knockdown significantly decreased the expression of PARP, caspase-3, cleaved caspase-3 and caspase-9 proteins in AsPC-1 (*p* < 0.05) and MiaPaCa-2 (*p* < 0.05) cells compared to the control cells. However, Bcl-2 expression increased significantly in AsPC-1 (*p* < 0.05) and MiaPaCa-2 (*p* < 0.01) cells after siRNA-PHB transfection (Fig. 3c). These data further support that the silencing of PHB expression reduces apoptosis in human pancreatic cancer cells via the mitochondrial pathway.

**Fig. 4:**
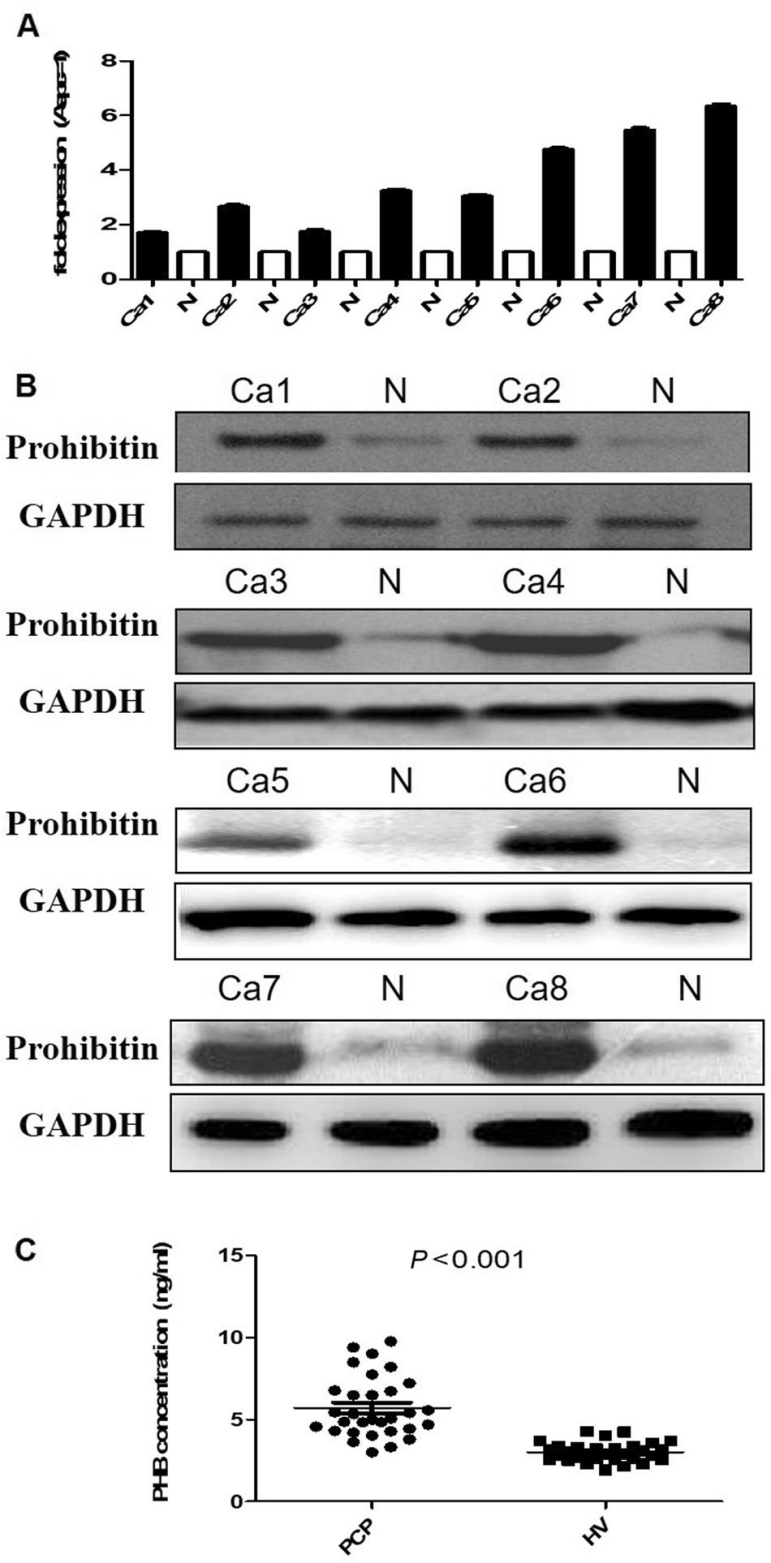
PHB higher expression in pancreatic cancer patients tissues and sera. **a** PHB mRNA expression was detected in 8 paired human pancreatic cancer tissues and normal pancreas with q-PCR analysis, the results were presented as the magnitude of relative expression (means ± SEM). β-actin was used as a housekeeping gene. **b** PHB protein expression was analysed in 8 paired human pancreatic cancer tissues and normal pancreas. Immunoblot analysis was performed using anti-rabbit antibodies (1:1000), GAPDH was used as a loading control. **c** The ELISA assay results showed that the level of serum PHB in the 31 PDAC patients was significant higher than that of 31 healthy volunteer (5.71 ± 1.85 ng/ml vs. 3.02 ± 0.59 ng/ml, *p* < 0.01)

### Obviously increased prohibitin expression in pancreatic cancer patient tissues and sera

PHB mRNA and protein were detected in 8 paired human pancreatic cancer tissues and normal pancreatic tissues using q-PCR and Western blot (clinical characteristics in Supplement Table 1). PHB mRNA and protein expression levels in the pancreatic cancer tissues were significantly increased compared to that in the normal pancreas samples (Fig. 4a, b). ELISAs demonstrated that the level of serum PHB in the 31 PDAC patients was significantly higher than that in the 31 healthy volunteers (5.71 ± 1.85 ng/ml vs. 3.02 ± 0.59 ng/ml, P < 0.001) (Fig. 4c) (Clinicopathological parameters in Supplement Table 2).

### Location, expression and clinicopathological relationship analyses of Prohibitin in pancreatic cancer tissues using IHC

**Fig. 5:**
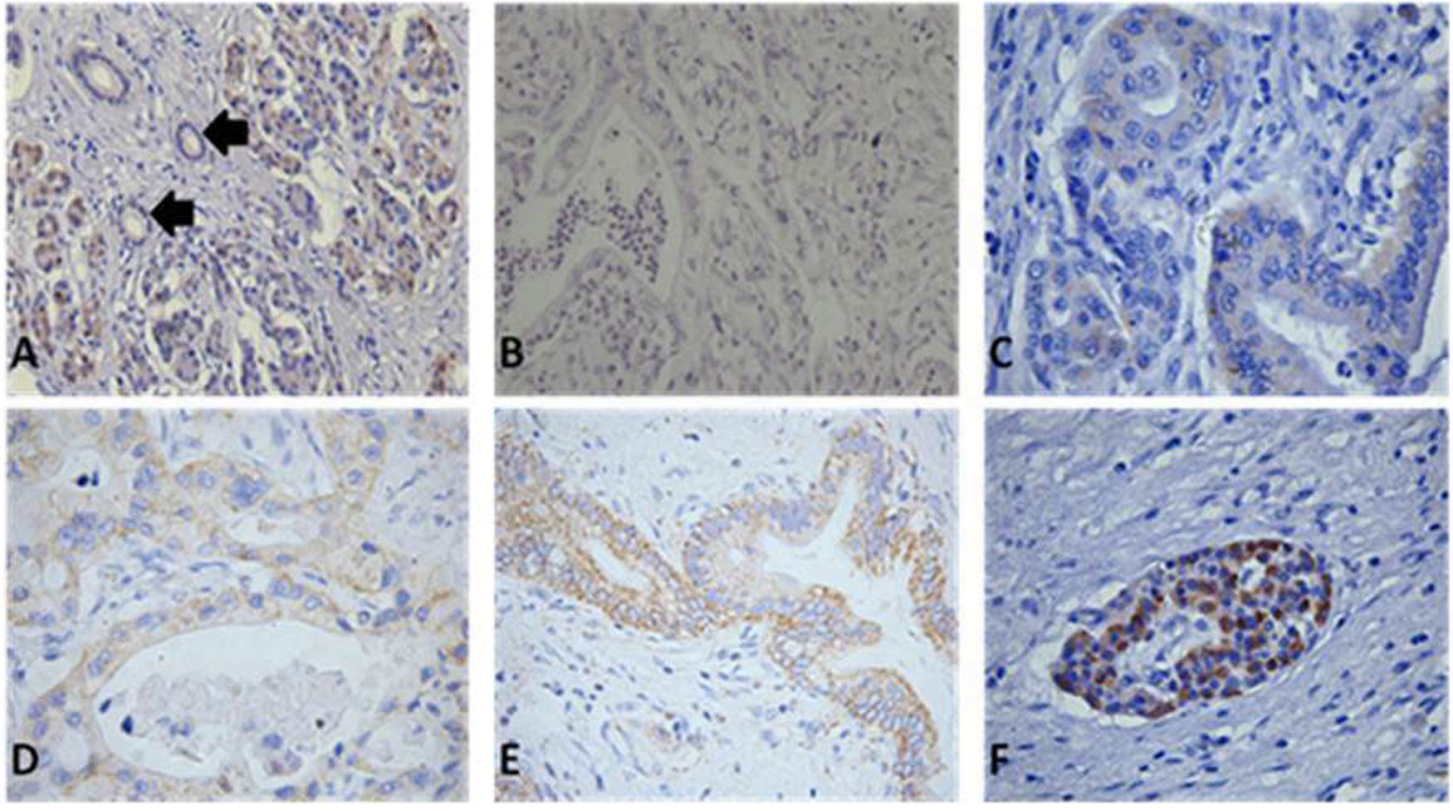
Location and expression of PHB in normal and cancerous pancreatic tissues. Immunohistochemistry was performed with PHB (1:5000) anti-rabbit antibodies. (magnitude ×200) **a** Localisation of PHB in normal pancreatic tissues, acinar cells and some ductal cells showed very weak cytoplasmic staining. **b** Very weak positive staining of PHB in cancer cells. **c** Weak expression of PHB shown by cytoplasmic staining. **d** Mild expression of PHB shown by plasma membrane and cytoplasm of cancer cells. **e** Strong expression of PHB shown by plasma membrane and cytoplasm of cancer cells. **f** PHB was also strong expressed in pancreatic islet cells

Immunohistochemistry was performed in 10 normal and 66 PDAC tissues. Acinar cells and some ductal cells in normal tissues exhibited very weak cytoplasmic staining (Fig. 5a). Strong PHB expression was observed in the plasma membrane and cytoplasm of cancer cells and degenerating acinar cells. Various intensities of PHB positivity were observed in cancer cells from the PDAC tissue samples (Fig. 5b–e). PHB was also strongly expressed in pancreatic islet cells (Fig. 5f). IHC revealed that 29 (43.9%) of 66 cases exhibited high PHB expression in the PDAC specimens, and the strong expression rate for para-pancreatic cancer tissues was only 14/66 (21.2%). These results suggest that PHB expression was significantly higher in PDAC tissues (*P* < 0.05). We further divided the PDAC patients into PHB-weak and PHB-strong expression groups based on the differential expression levels of PHB in PDAC tissues. Table 1 shows the demographics and correlations between PHB expression and clinicopathological features. The rate of distant metastasis in follow-up was significant higher in the high PHB expression group than the low PHB expression group (79.3 vs. 35.1%) (*P* < 0.001), which indicates that the overexpression of PHB was associated with the progression of pancreatic cancer.

**Table 1:** Correlation of PHB expression with clinicopathologic parameters

clinicopathologic Characteristics	No. of patients	Prohibitin expression		*P* value
		High	Low	
Overall frequency				0.009**
PC tissure	66	29	37	
Normal tissure	66	14	52	
Age				1
<65	43	19	24	
≥65	23	10	13	
Gender				0.153
Male	35	12	23	
Female	31	17	14	
Tumour location				0.314
Head	42	16	26	
Body/tail	24	13	11	
Histological grade^a^				0.705
Grade 1/2	37	15	22	
Grade 3	29	14	15	
Pathological T stage				0.375
T1/T2	11	3	8	
T3/T4	55	29	37	
Lymph node involvement				0.402
−	30	11	19	
+	36	18	18	
TNM stage				0.443
I	5	1	4	
II	48	23	25	
III/IV	13	5	8	
Distant metastasis in follow-up				<0.001**
−	30	6	24	
+	36	23	13	

** *P* < 0.01

^a^Grade 1, well differentiated; Grade 2, moderately differentiated; Grade 3, poorly differentiated

### Survival analysis and univariate/multivariate analyses of the prognostic value of Prohibitin expression in pancreatic cancer

**Fig. 6:**
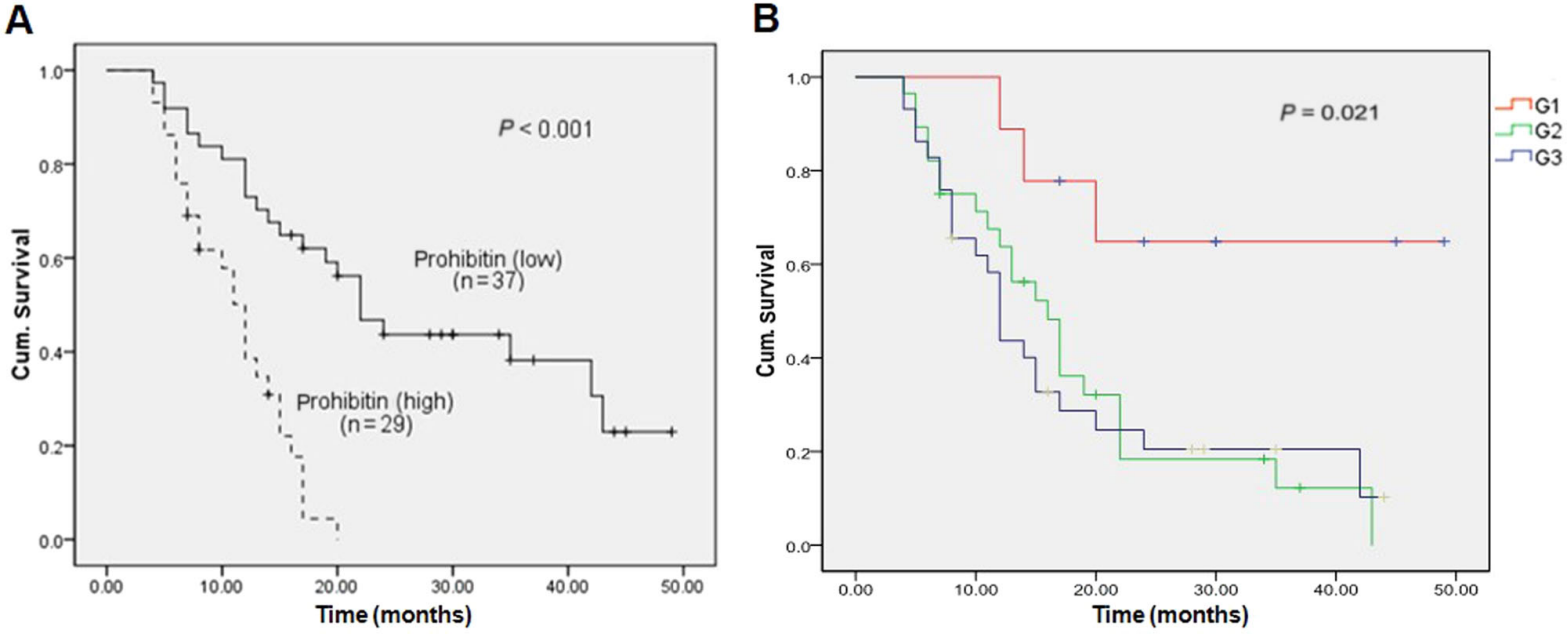
Kaplan–Meier and log-rank analyses of prognostic factors assessed in multivariable analysis using a COX proportional hazards model. Median values were taken as cutoff limits when the two groups were compared. Survival analyses were performed using the Kaplan–Meier method for the estimation of event rates, and a log-rank test was used for survival comparisons between patient groups. The *P* value was 5%. PHB expression level (*p* < 0.01) and histological G stage (*p* < 0.05) both were independent prognostic factors, but the others risk factors, although showing the classical tendencies, did not appear to impact on the prognosis independency. (figures not showed)

Patients with lower levels of PHB expression [*n* = 37, median survival = 27.4 (21.9–33.1) months] exhibited significantly longer survival times compared to patients with higher PHB expression [*n* = 29, median survival = 11.1 (9.4–12.9) months, *p* < 0.01] (Fig. 6a). The prognostic value of PHB expression was assessed using a Cox proportional hazards model, and the results revealed that PHB expression (HR = 4.25, 95% CI: 2.09–4.26, *P* < 0.01) and histological grade (HR = 1.67, 95% CI: 1.07–2.16, *P* < 0.05) were independent prognostic factors (Fig. 6b). Other known prognostic factors (e.g., TNM stage and lymph node involvement) did not impact the prognosis independently because of the scarcity of early and less aggressive tumours in our cohort of 66 patients (Table 2).

**Table 2:** Univariable and multivariable analysis of prognostic factors

	Univariable analysis					Multivariable analysis		
	Comparator	*N*	Median survival (95% CI) in months	Log-rank	*p* value	HR	95% CI	*p* value
Median age (years)	≥65.5 vs. <65.5	23/43	18.2 (12.8–23.7) vs. 21.6 (16.6–26.6)	0.291	0.508	1.811	0 0.307–0.791	0.325
Gender	M/F	35/31	18.9 (13.7–24.1) vs. 22.1 (16.8–27.4)	0.575	0.239	1.158	0.297–1.463	0.589
Tumour location	Head vs. body/tail	42/24	22.5 (17.4–27.5) vs. 17.5 (11.7–23.4)	0.436	0.174	0.976	0.398–1.324	0.435
T-status	T1/T2 vs. T3/T4	11/55	29.2 (19.8–38.7) vs. 18.9 (14.8–23.1)	0.194	0.097	1.576	0.12–2.312	0.292
N-status	N0 vs. N1	30/36	25.6 (19.5–31.8) vs.16.2 (12.0–20.5)	0.163	0.016	1.18	0.63–2.21	0.61
TNM-stage	I/II/III/IV	5/48/6/7	30.1 (22.4–37.6) vs.19.3 (15.3–23.3) vs.26.7 (13.5–39.8) vs.10.4 (2.7–18.2)	0.114	0.025	1.12	0.53–2.36	0.76
Grade	G1 vs. G2 vs. G3	9/28/29	37.2 (26.4–48.1) vs. 18.1 (13.4–22.9) vs.17.6 (12.4–22.7)	0.51	0.021	1.67	1.07–2.61	0.026*
PHB score	≤4 vs. >4	37/29	27.4 (9.4–12.9) vs. 11.1 (21.9–33.0)	1.446	0.001	4.25	2.09–4.26	0.001**

^*^p < 0.05, **p < 0.01

## Discussion

Recent studies reported that PHB was overexpressed in several tumour types, and it plays crucial roles in cancer development and progression, including hepatocellular carcinoma, mammary cancer, squamous cell carcinoma of the lung and gastric cancer^16–19^. PHB is also overexpressed in gallbladder cancer, and it predicts poor prognosis and promotes cell proliferation and invasion^20^. Our study detected PHB mRNA and protein expression in 8 paired pancreatic cancer and normal pancreas tissues, and the expression of both entities were obviously higher in pancreatic cancer tissues than normal pancreas. The IHC results demonstrated higher PHB expression in 66 pancreatic cancer tissues compare to para-cancer tissues. We also found that higher PHB expression predicted a shorter overall survival time based on survival analysis. PHB and pathological stage G were independent risk factors in multivariate analyses using a Cox proportional hazards model, which suggests that PHB may be used as a potential factor for survival. Mengwasser et al. reported obviously higher PHB expression levels in colorectal cancer patient sera than normal sera^21^. PHB levels in our study were significantly higher in pancreatic cancer patient sera than in healthy volunteer sera according to ELISA. Certainly, in our study, the sample size was small, and we only compared healthy volunteer and pancreatic cancer patients, this result had its own limitations. Next step, we will enlarge sample size and include more category of pancreatic and other digestive system diseases to identify this reselt deeply, but now, which means that PHB may be a new biomarker in pancreatic cancer patients in some extent.

PHB is a hub for many signalling pathways triggered by growth factors, the immune response, steroid hormones that regulate metabolism, and mitochondrial biogenesis. PHB is involved in growth, resistance to chemotherapy, and metastasis via several mechanisms, including activation of the Ras-C-Raf-MEK-ERK pathway, modulation of TGF-β signalling, and regulation of transcription^22^. Many studies have demonstrated that PHB played an important role in the activation of the PI3K/Akt and Ras-C-Raf-MEK-ERK signalling pathways, in the modulation of epithelial cell adhesion and migration, and in the promotion of cancer metastasis^23,24^. Yang Cao et al. reported that PHB promoted cell proliferation and invasion via ERK pathway activation in gallbladder cancer^20^. Zhou Luan et al. reported that blockade of the PHB scaffold-CRAF kinase interaction, which is distinct from direct kinase inhibition, may be a new therapeutic strategy to target oncogenic ERK-driven pancreatic cancers^25^. Our study verified that the proliferation, migration and invasion abilities were significantly inhibited in PHB under-expressing pancreatic cancer cells. In contrast, the proliferation, migration and invasion abilities were up-regulated in PHB over-expressing pancreatic cancer cells.

PHB is a small conserved protein that is implicated in numerous functions in mitochondria^26^. PHB appears indispensable for mitochondrial translation, and its ablation leads to a decrease in mitochondrial membrane potential^27,28^. Long Zhang et al. reported increased BAX and Cyt.c expression in PHB over-expressing gastric cancer BGC823 cells and decreased Bcl-2 expression. The activation levels of caspase-3 and caspase-9 were increased, but the activation level of caspase-8 was not changed, which suggests that PHB-induced apoptosis via the mitochondrial pathway^29^. Consistent with our study results, the expression of PARP, cleaved caspase-3, and caspase-9 was obviously decreased in PHB under-expressing pancreatic cancer cell lines AsPC-1 and MiaPaCa-2, but Bcl-2 was apparently increased. The activation level of caspase-3 was also decreased, which indicates that siRNA-PHB was an inhibitor of apoptosis in pancreatic cancer cells. Bcl-2 family proteins play an important role in regulating mitochondrial function and apoptosis. Bcl-2 family proteins form pore compounds in the mitochondrial inter membrane and release apoptotic factors into the cytoplasm to cause apoptosis^30^. The exact mechanism of PHB in apoptosis in different cancer cells is not clear. Yingjie Xu et al. reported that PHB1 regulates tumour cell apoptosis via interaction with X-linked inhibitor of apoptosis protein^31^. Sanchez-Quiles V et al. reported that PHB regulates apoptosis in a mechanism dependent on NFκB signalling in human hepatoma cells^32^.

In summary, we demonstrated that high PHB expression in pancreatic cancer tissues and pancreatic cancer patient sera was associated with poor prognosis in pancreatic cancer patients. Furthermore, down-regulation of PHB expression reduced the proliferation, migration and invasion of pancreatic cancer cells, and the opposite results were obtained in PHB over-expressing cells. Therefore, PHB may be a novel prognosis marker and candidate for targeted therapy against pancreatic cancer.

### Study Highlights

#### 1. WHAT IS CURRENT KNOWLEDGE

- More than one-half of pancreatic cancer cases are diagnosed at a distant stage.
- Novel specific markers in pancreatic cancer patients are strong demanded.

#### 2. WHAT IS NEW HERE

- PHB is significantly increased in PDAC tissues and patients sera.
- PHB is an independent prognostic factor in PDAC patients.
- PHB plays a key role in modulating the malignant phenotype and apoptosis induction.

#### 3. TRANSLATIONAL IMPACT

- PHB may be a novel prognostic predictor and a candidate for targeted therapy against PDAC.

## Electronic supplementary material

SupplementaryTable 2

SupplementaryTable 1
