# Supplementary material for: A novel prognostic marker and immunogenic membrane antigen: prohibitin (PHB) in pancreatic cancer
Source: Clin Transl Gastroenterol. 2018 Sep 6;9(9):178. doi: 10.1038/s41424-018-0044-1 (PMC6125288; doi:10.1038/s41424-018-0044-1)
Supplement: Supplementary file 1 — SupplementaryTable 2 [file 41424_2018_44_MOESM1_ESM.docx]

**Supplement Table 2. Correlation of PHB expression in pancreatic patients’ sera with clinicopathologic parameters**

| **Variables** | **N** | **Mean ± SE(ng/mL)** | ***t*** | ***P* value** |
| --- | --- | --- | --- | --- |
| **Histological grade^a^** |  |  | 1.39 | 0.176 |
| Grade 1/2 | 19 | 5.35±1.78 |  |  |
| Grade 3 | 12 | 6.28±1.89 |  |  |
| **Pathological T stage** |  |  | -0.967 | 0.342 |
| T1/T2 | 2 | 4.49±0.30 |  |  |
| T3/T4 | 29 | 5.79±1.88 |  |  |
| **Lymph node involvement** |  |  | -0.373 | 0.712 |
| － | 12 | 5.55±1.63 |  |  |
| ＋ | 19 | 5.58±2.01 |  |  |
| **TNM stage** |  |  | 0.018 | 0.986 |
| Ⅰ/Ⅱ | 19 | 5.71±1.83 |  |  |
| Ⅲ/Ⅳ | 12 | 5.70±1.95 |  |  |

^a^Grade 1,well differentiated; Grade 2, moderately differentiated; Grade 3, poorly differentiated
