# Supplementary material for: A novel prognostic marker and immunogenic membrane antigen: prohibitin (PHB) in pancreatic cancer
Source: Clin Transl Gastroenterol. 2018 Sep 6;9(9):178. doi: 10.1038/s41424-018-0044-1 (PMC6125288; doi:10.1038/s41424-018-0044-1)
Supplement: Supplementary file 2 — SupplementaryTable 1 [file 41424_2018_44_MOESM2_ESM.doc]

| **Variables** | **No. of patients** |
| --- | --- |
| **Gender**  Male | 3 |
| Female | 5 |
| **Age (years)** | Median = 60.3 |
| **Pathological T stage**  T1& T2 | 3 |
| T3a | 5 |
| **Lymph node involvement**  N0 | 2 |
| N1 | 6 |
| **Histological grade^a^**  G1a | 0 |
| G2 | 4 |
| G3 | 4 |

**Supplement Table.1 Data of 8 paired pancreatic cancer patients’ characteristics**

^a^Grade 1,well differentiated; Grade 2, moderately differentiated; Grade 3, poorly differentiated
